# Supplementary material for: Multi-ancestry investigation of the genomics of erectile dysfunction
Source: Nat Commun. 2025 Nov 24;16:11602. doi: 10.1038/s41467-025-66723-7 (PMC12749275; doi:10.1038/s41467-025-66723-7)
Supplement: Supplementary file 1 — Supplementary Information [file 41467_2025_66723_MOESM1_ESM.pdf]

Supplementary Figures 1-5

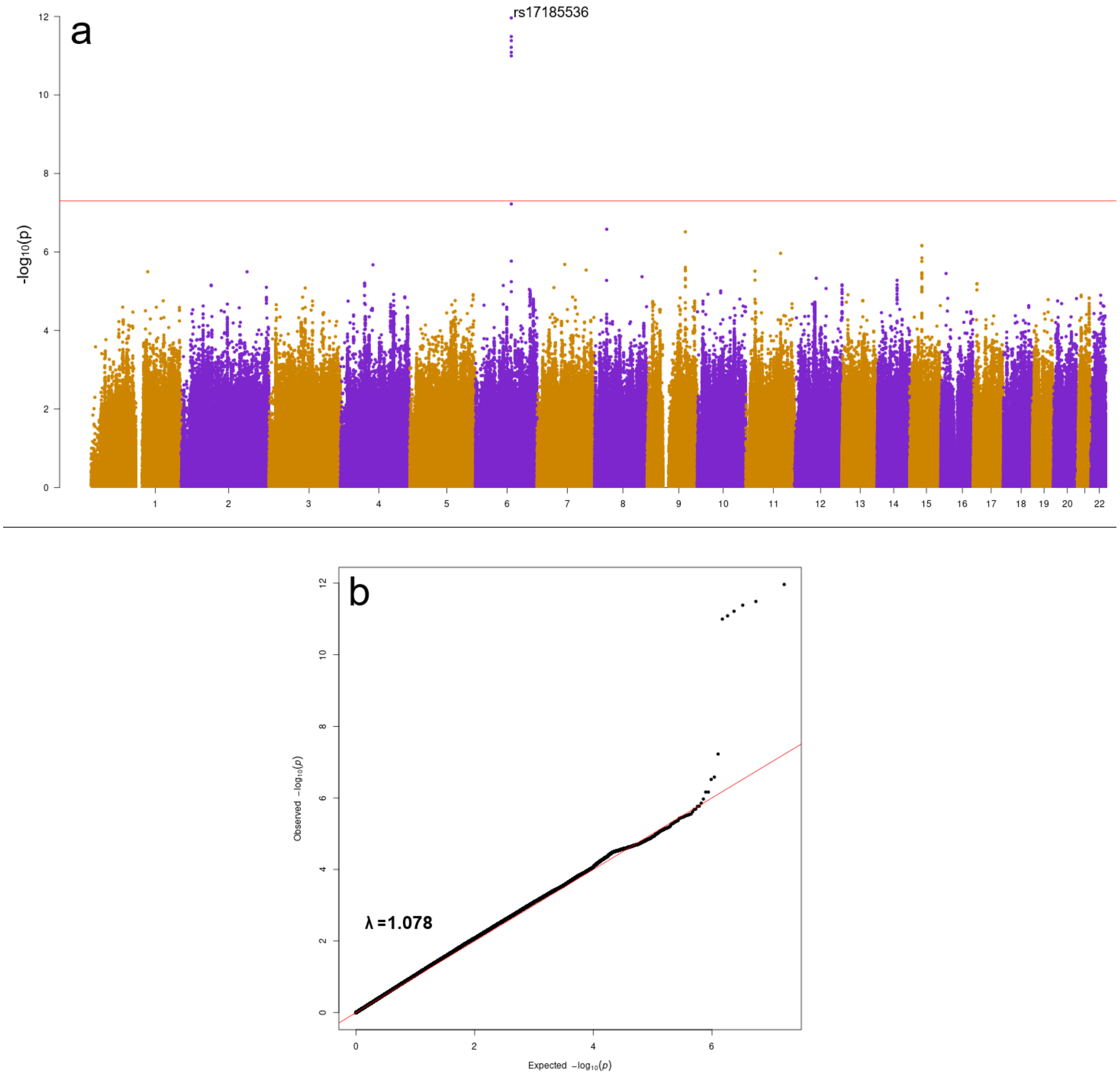

Supplementary Figure 1: (a) GWAS and **(b)** Quantile-quantile (QQ) plot of EHR-defined erectile dysfunction (EHR-ED) in EUR population from the All of Us biobank (n_{total}=88,722, n_{eff}=54,929).

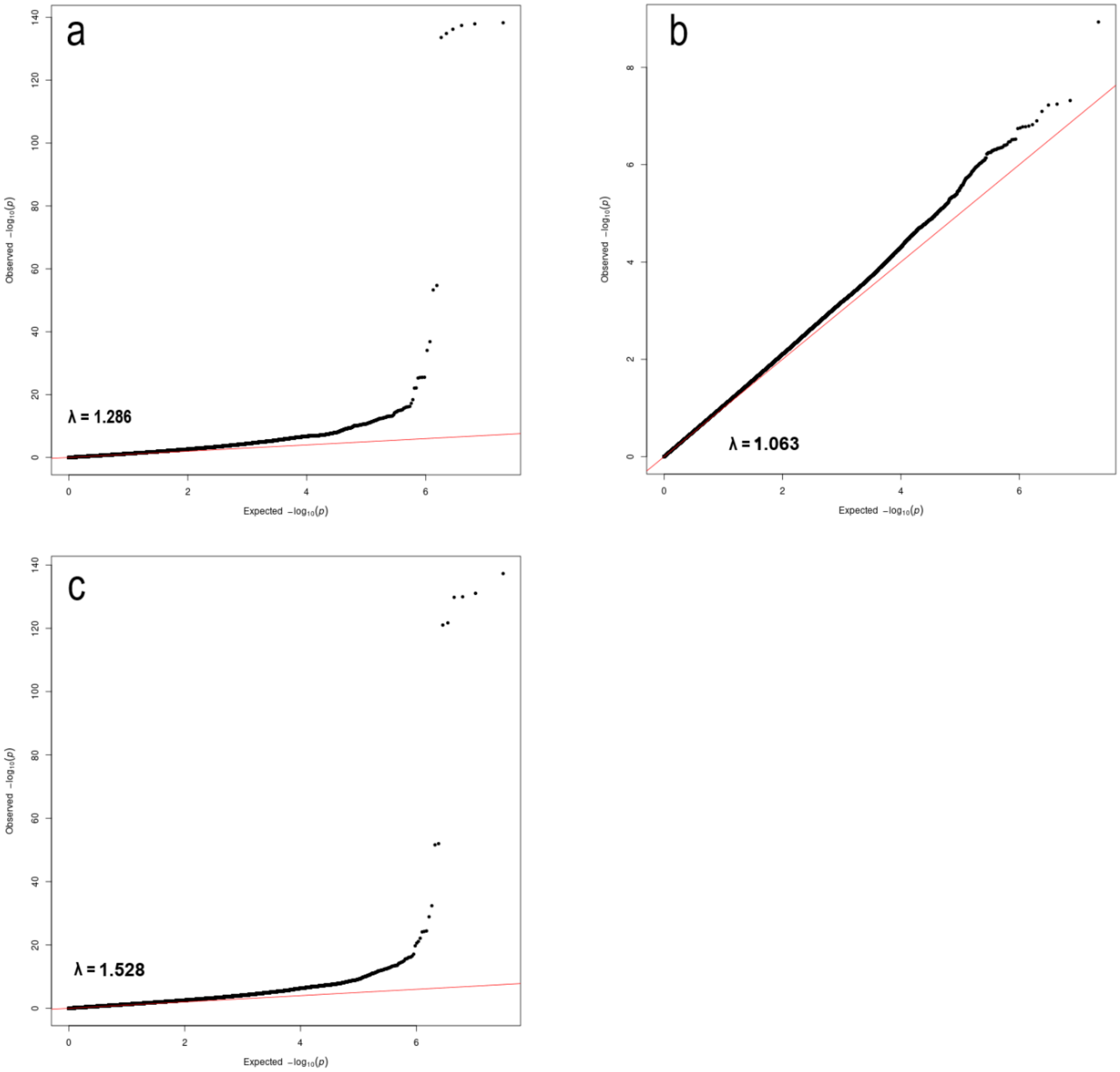

Supplementary Figure 2: Quantile-quantile (QQ) plot of EHR-defined erectile dysfunction (EHR-ED) in **(a)** EUR ancestry, **(b)** AFR ancestry, and **(c)** cross-ancestry (EUR-AFR).

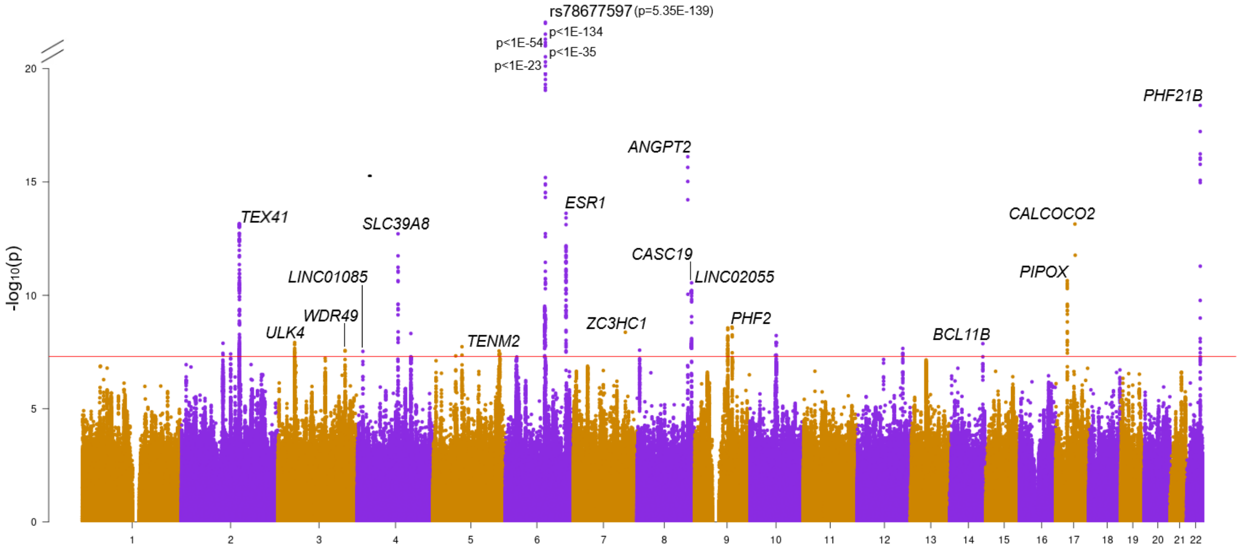

Supplementary Figure 3: GWAS meta-analysis of EHR-defined erectile dysfunction (EHR-ED) in EUR population (truncated Manhattan plot). Variants that are located within or near genes are annotated (n_{total}=913,194, n_{eff}=465,415) Statistical significance is defined by the standard p-value threshold of $5x10^{-8}$

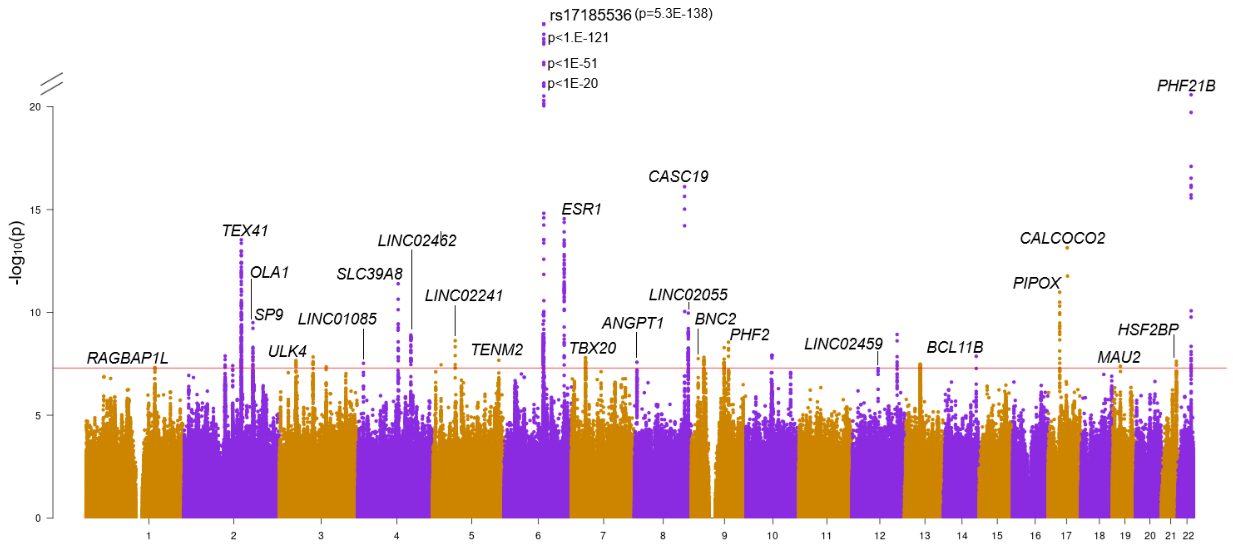

Supplementary Figure 4: GWAS meta-analysis of EHR-defined erectile dysfunction (EHR-ED) in cross-ancestral (EUR-AFR) analysis (truncated Manhattan plot). Variants that are located within or near genes are annotated n_{total}=1,038,509, n_{eff}=617,054). Statistical significance is defined by the standard p-value threshold of $5x10^{-8}$

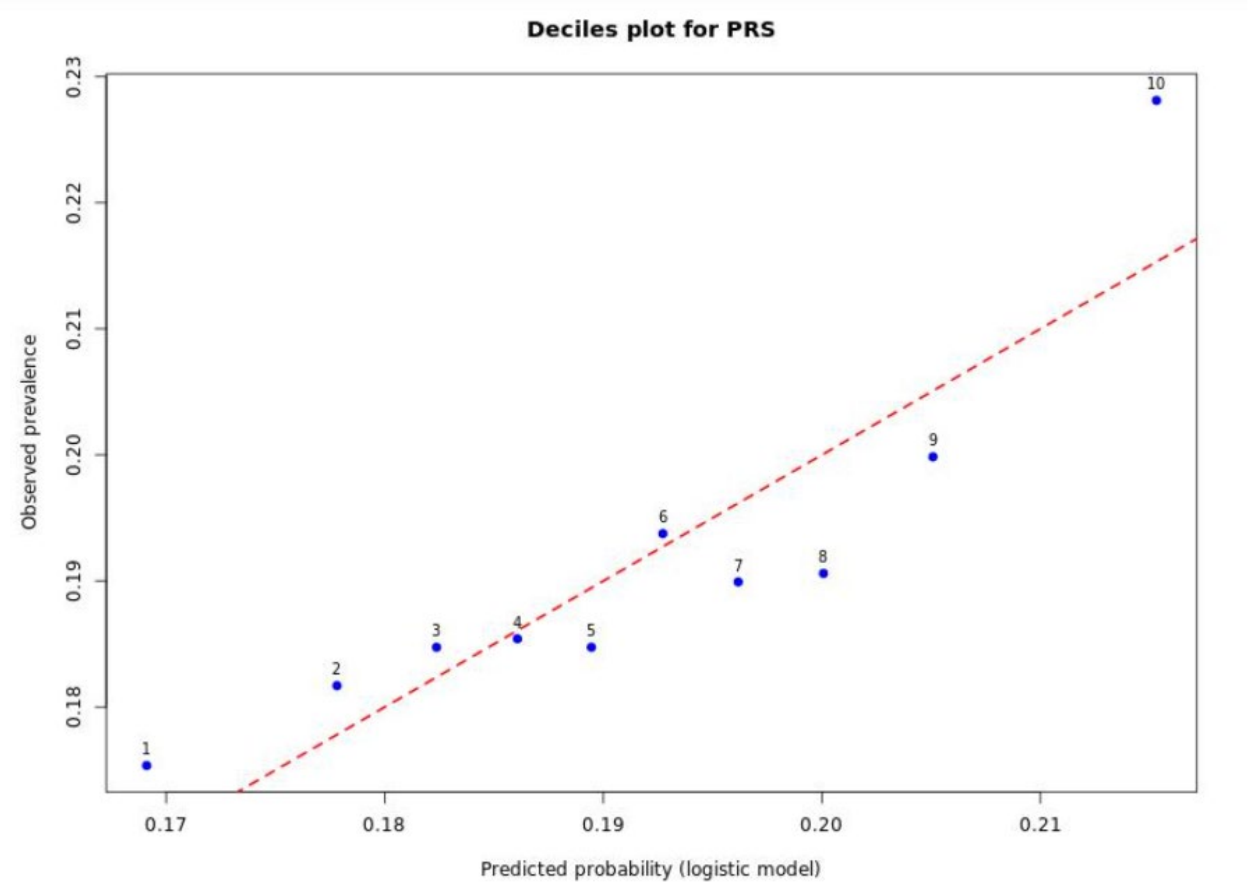

Supplementary Figure 5: EHR-ED predicted probability (based on EHR-ED PRS with a significance threshold of p=0.5, using a leave-AoU-out approach) vs EHR-ED prevalence in AoU.

Supplementary Tables 1-9

Supplementary Table 1: Demographic characteristics of AoU participants included in the study

		Cases	Controls	Total
**EUR**	Mean Age (± SD)	66 (±12)	56 (± 17)	58 (± 17)
	Mean BMI (± SD)	29.3 (±5.1)	28.7 (±5.4)	28.9 (± 5.3)
	History of prostate cancer*	17.76%	5.30%	7.69%
	Type 2 Diabetes*	31.04%	14.81%	17.91%
**AFR**	Mean Age (± SD)	59 (±11)	49 (±14)	50 (± 14)
	Mean BMI (± SD)	29.9 (± 5.8)	28.2 (± 6.1)	28.9 (± 6)
	History of prostate cancer*	16.04%	1.91%	3.87%
	Type 2 Diabetes*	50.84%	12.73%	17.77%

*Self report + EHR

Supplementary Table 2: Heritability estimates across the various EUR cohorts

Ancestry	**Cohort**	**h2**
EUR	**AoU**	0.034 (0.011)
	**Finngen**	0.233 (0.064)
	**Bovijn et al 2019 ***	0.089 (0.024)
	**Verma et al 2024 ****	0.063 (0.003)
	**Total (Meta-Analysis)**	0.062 (0.004)

* UK Biobank; Estonian Genome Center of the University of Tartu; Partners HealthCare Biobank. PMID: 30583798

** MVP. PMID: 39024449

Supplementary Table 3: Inter-cohort -EHR-ED genetic correlations (LDSC)

	**AoU**	**Bovijn 2019 ***	**Verma et al 2024 ****
**Bovijn 2019 ***	rg=0.289 (0.213), p=0.176 (ns)		
**Verma et al 2024 ****	rg=0.506 (0.136), p=0.0002	rg=1.017 (0.157), p=9.16E-11	
**Finngen**	rg=0.481 (0.215), p=0.025	rg=0.2721 (0.1898), p=0.15 (ns)	rg= 0.539 (0.103), p=1.62E-07

* UK Biobank; Estonian Genome Center of the University of Tartu; Partners HealthCare Biobank. PMID: 30583798

** MVP. PMID: 39024449

Supplementary Table 4: PRS prediction of EHR-ED. A leave-one-out approache, excluding AoU data from the meta analysis and using it a target sample (two-sided logistic regression).

Ancestry	p-value threshold	Estimate	P value	Nagelkerke r2
EUR	0.000001	2.60	3.10E-04	0.0899
	0.00001	2.60	3.10E-04	0.0899
	0.0001	2.65	6.10E-02	0.0897
	0.001	8.79	8.00E-05	0.0899
	0.01	21.43	3.10E-02	0.0897
	0.05	202.58	9.70E-07	0.0901
	0.1	473.64	1.60E-09	0.0903
	0.2	1401.86	1.60E-20	0.0911
	0.3	2347.12	7.20E-26	0.0916
	0.4	3482.36	3.20E-32	0.0921
	**0.5**	**4612.40**	**2.20E-36**	**0.0924**
AFR	0.000001	-1.31	1.70E-01	0.1278
	0.00001	9.34	2.20E-02	0.128
	0.0001	35.96	3.20E-03	0.1282
	0.001	88.32	2.20E-02	0.128
	0.01	266.21	1.70E-02	0.128
	0.05	574.00	8.70E-03	0.1281
	0.1	826.00	5.70E-03	0.1281
	**0.2**	1214.77	3.70E-03	0.1282
	0.3	1480.96	4.30E-03	0.1282
	0.4	1692.84	5.60E-03	0.1281
	0.5	1913.42	6.00E-03	0.1281

Supplementary Table 5: Sensitivity and Specificity of PRS prediction of EHR-ED. A leave-one-out approach, excluding AoU data from the meta analysis and using it a target sample.

		Observed		
		**Controls**	**Cases**	**Sensitivity**
Predicted	**Controls**	54,156	12,286	0.28
	**Cases**	17,583	4,697	
	**Specificity**	0.75		

Supplementary Table 6: TWAS (FUSION)

Tissue	Gene ID	Gene Symbol	TWAS.Z	TWAS.P
Brain Amygdala	ENSG00000168036.16	*CTNNB1*	-5.8	6.80E-09
Pituitary	ENSG00000275120.2		5.8	8.00E-09
Lung	ENSG00000137103.16	*TMEM8B*	-5.6	2.20E-08
Stomach	ENSG00000163536.12	*SERPINI1*	5.6	2.30E-08
Esophagus Gastroesophageal Junction	ENSG00000106392.10	*C1GALT1*	5.6	2.40E-08
Testis	ENSG00000226674.8	*TEX41*	-5.5	3.20E-08
Brain Cerebellar Hemisphere	ENSG00000228566.1	*LOC124902438, LOC124902439*	5.5	4.20E-08
Cells Cultured Fibroblasts	ENSG00000110514.19	*MADD*	5.3	1.10E-07
Ovary	ENSG00000165282.13	*PIGO*	5.3	1.30E-07

Supplementary Table 7: Drugs Repuposing (drug.MATADOR, ShinyGO 0.82)

Enrichment FDR	nGenes	Pathway Genes	Fold Enrichment	Pathway	Genes
0.027748594	1	5	176.008	Fulvestrant	*ESR1*
0.027748594	1	7	125.720	Sulindac	*CTNNB1*
0.027748594	1	3	293.346	Phenol red	*ESR1*
0.027748594	1	6	146.673	Danazol	*ESR1*
0.04876932	1	20	44.002	Implanon	*ESR1*
0.04876932	1	18	48.891	Tibolone	*ESR1*
0.04876932	1	25	35.202	Raloxifene	*ESR1*
0.04876932	1	28	31.430	Estrogen	*ESR1*
0.04876932	1	23	38.263	Diethylstilbestrol	*ESR1*
0.060780678	1	43	20.466	Toremifene	*ESR1*
0.060780678	1	43	20.466	Thalidomide	*ANGPT2*
0.088161748	1	75	11.734	Tamoxifen	*ESR1*
0.088161748	1	73	12.055	Spironolactone	*ESR1*
0.101557131	1	94	9.362	Mifepristone	*ESR1*

Supplementary Table 8: gSEM: exploratory factor analysis (EFA)

Loadings:

Trait	Factor 1	Factor 2	Factor 3
ADHD	0.307	0.442	0.183
CanUD	0.962		
Depression		0.916	
ED	0.323		0.298
Endometriosis		0.255	
HighBP			0.627
IschaemicHD			0.601
SexPartners	0.571		
Obesity	-0.118		0.583
OUD	1.018		
PAU	0.8		-0.118
PTSD		1.078	
T2D		-0.117	0.855

	Factor 1	Factor 2	Factor 3
SS loadings	3.163	2.292	1.978
Proportion Var	0.243	0.176	0.152
Cumulative Var	0.243	0.42	0.572
