# Supplementary material for: Multi-ancestry investigation of the genomics of erectile dysfunction
Source: Nat Commun. 2025 Nov 24;16:11602. doi: 10.1038/s41467-025-66723-7 (PMC12749275; doi:10.1038/s41467-025-66723-7)
Supplement: Supplementary file 2 — Description of Additional Supplementary Files [file 41467_2025_66723_MOESM2_ESM.pdf]

Description of Additional Supplementary Files

Supplementary Data 1: Lead SNPs

Supplementary Data 2: Gene-based analysis (MAGMA) in EUR ancestry.

Supplementary Data 3: Gene-based analysis (MAGMA) in AFR ancestry.

Supplementary Data 4: Gene-based analysis (MAGMA) in the cross-ancestry analysis.

Supplementary Data 5: Genetic correlations between EHR-ED and selectEHR-ED traits (LDSC)

Supplementary Data 6: Genetic correlations between EHR-ED and 3,935 brain measures

Supplementary Data 7: Local genetic correlations between EHR-ED and selected traits (LAVA)

Supplementary Data 8: Mendelian randomization (MR) results with EHR-ED as exposure

Supplementary Data 9: Mendelian randomization (MR) results with EHR-ED as outcome

Supplementary Data 10: pheWAS of rs78677597, in 330 traits in Finngen, UKBB and MVP (https://mvp-ukbb.finngen.fi). Significant values are written in bold letters.

Supplementary Data 11: Summary-based Mendelian Randomization (SMR) results

Supplementary Data 12: Drug Repurposing - List of tesed genes

Supplementary Data 13: gSEM: confirmatory factor analysis (CFA)
