# Supplementary material for: Multi-ancestry investigation of the genomics of erectile dysfunction
Source: Nat Commun. 2025 Nov 24;16:11602. doi: 10.1038/s41467-025-66723-7 (PMC12749275; doi:10.1038/s41467-025-66723-7)
Supplement: Supplementary file 4 — Reporting Summary [file 41467_2025_66723_MOESM4_ESM.pdf]

nature portfolio

Corresponding author(s): Joel Gelernter

Last updated by author(s): Jul 15, 2025

Reporting Summary

Nature Portfolio wishes to improve the reproducibility of the work that we publish. This form provides structure for consistency and transparency in reporting. For further information on Nature Portfolio policies, see our Editorial Policies and the Editorial Policy Checklist.

Statistics

For all statistical analyses, confirm that the following items are present in the figure legend, table legend, main text, or Methods section.

n/a	Confirmed	
☐	☒	The exact sample size (n) for each experimental group/condition, given as a discrete number and unit of measurement
☒	☐	A statement on whether measurements were taken from distinct samples or whether the same sample was measured repeatedly
☐	☒	The statistical test(s) used AND whether they are one- or two-sided<br>*Only common tests should be described solely by name; describe more complex techniques in the Methods section.*
☐	☒	A description of all covariates tested
☐	☒	A description of any assumptions or corrections, such as tests of normality and adjustment for multiple comparisons
☐	☒	A full description of the statistical parameters including central tendency (e.g. means) or other basic estimates (e.g. regression coefficient) AND variation (e.g. standard deviation) or associated estimates of uncertainty (e.g. confidence intervals)
☐	☒	For null hypothesis testing, the test statistic (e.g. F, t, r) with confidence intervals, effect sizes, degrees of freedom and P value noted<br>*Give P values as exact values whenever suitable.*
☒	☐	For Bayesian analysis, information on the choice of priors and Markov chain Monte Carlo settings
☒	☐	For hierarchical and complex designs, identification of the appropriate level for tests and full reporting of outcomes
☒	☐	Estimates of effect sizes (e.g. Cohen's d, Pearson's r), indicating how they were calculated

Our web collection on statistics for biologists contains articles on many of the points above.

Software and code

Policy information about availability of computer code

Data collection	n/a
Data analysis	We used the following software: PLINK 2.0 for GWAS, METAL (version March 3, 2011) for meta-analyses, X, FUMA 1.5 and MAMGA (implemented in FUMA) for post-GWAS analyses, LDSC v1.0.1 for SNP-based heritability and genetic correlation analyses, LAVA for local genetic correlations, MRlap for Mendelian Randomization, FUSION (version May 24, 2022) for TWAS, genomic-SEM v0.05 for genomic structural equation modeling, PLINK 1.9 for PRS, drug.MATADOR (implemented in ShinyGO 0.82) for drug repurposing analysis.<br>All software packages used in this analysis are publicly available.

For manuscripts utilizing custom algorithms or software that are central to the research but not yet described in published literature, software must be made available to editors and reviewers. We strongly encourage code deposition in a community repository (e.g. GitHub). See the Nature Portfolio guidelines for submitting code & software for further information.

Data

Policy information about availability of data

All manuscripts must include a data availability statement. This statement should provide the following information, where applicable:

- Accession codes, unique identifiers, or web links for publicly available datasets
- A description of any restrictions on data availability
- For clinical datasets or third party data, please ensure that the statement adheres to our policy

All MVP and meta-analysis summary statistics are made available through CIPHER https://phenomics.va.ornl.gov/web/cipher/partner/mvp: email CIPHER@va.gov

with title "MVP and meta-analyses Summary Results – Galimberti et al".

Research involving human participants, their data, or biological material

Policy information about studies with human participants or human data. See also policy information about sex, gender (identity/presentation), and sexual orientation and race, ethnicity and racism.

Reporting on sex and gender	The study was of a male-limited trait, therefore all participants affected with this trait are males. Comparisons are made for numerous traits that include females. We have no available information on gender.
Reporting on race, ethnicity, or other socially relevant groupings	GWAS were conducted according to ancestry (European, African).
Population characteristics	GWAS were conducted on male European and African participants in the All of Us biobank, and meta-analyzed with available summary statistics from other studies.
Recruitment	All of Us participants enrolled digitally online, completed baseline health surveys and provided access to their electronic health records (EHR). They then proceeded to physical examination and provided biospecimen. The procedure is described in detail in PMID 31412182.<br>For all datasets used in this study other than All of Us, we did not access individual participants' data, and only used publicly-available summary statistics.
Ethics oversight	All of Us is supervised by the All of Us IRB (AoUIRBContact@emmes.com). At the Yale site, we used only de-identified data.

Note that full information on the approval of the study protocol must also be provided in the manuscript.

Field-specific reporting

Please select the one below that is the best fit for your research. If you are not sure, read the appropriate sections before making your selection.

☒ Life sciences ☐ Behavioural & social sciences ☐ Ecological, evolutionary & environmental sciences

For a reference copy of the document with all sections, see nature.com/documents/nr-reporting-summary-flat.pdf

Life sciences study design

All studies must disclose on these points even when the disclosure is negative.

Sample size	Sample size reflected our best efforts to gather all possible participants with genetic data and available phenotypes as described. We did not pre-determine sample sizes.
Data exclusions	We excluded individuals without all the necessary information to run the GWAS analyses. We excluded all females, since ED is a male-specific trait.
Replication	GWAS summary statistics are available for download in our lab website.
Randomization	Observational study - not applicable
Blinding	Data were collected entirely independently of the analysts. There was no need for blinding or randomization.

Reporting for specific materials, systems and methods

We require information from authors about some types of materials, experimental systems and methods used in many studies. Here, indicate whether each material, system or method listed is relevant to your study. If you are not sure if a list item applies to your research, read the appropriate section before selecting a response.

Materials & experimental systems

n/a	Involved in the study	
☒	☐	Antibodies
☒	☐	Eukaryotic cell lines
☒	☐	Palaeontology and archaeology
☒	☐	Animals and other organisms
☒	☐	Clinical data
☒	☐	Dual use research of concern
☒	☐	Plants

Methods

n/a	Involved in the study	
☒	☐	ChIP-seq
☒	☐	Flow cytometry
☒	☐	MRI-based neuroimaging

Plants

Seed stocks	NA
Novel plant genotypes	NA
Authentication	NA
